# Topology-Guided Multi-Level Graph Learning: A Unified Framework for Molecular Property Prediction

**DOI:** 10.3390/molecules31193436

**Published:** 2026-09-27

**Authors:** Hasnain Ali, Momina Arshad, Malik Ahsin Iqbal, Motasem S. Alsawadi, Fahad Alghannam

**Affiliations:** 1 Abdus Salam School of Mathematical Sciences, Government College University, Lahore 54600, Pakistan; hasnain.ali_22@sms.edu.pk (H.A.); momina.arshad_22@sms.edu.pk (M.A.); malikahsin34@gmail.com (M.A.I.); 2 Institute of Next Generation Connectivity, King Abdulaziz City for Science and Technology (KACST), Riyadh 12354, Saudi Arabia; 3 Microelectronics and Semiconductors Institute, King Abdulaziz City for Science and Technology (KACST), Riyadh 12354, Saudi Arabia

**Keywords:** molecular property prediction, graph neural networks, graph transformers, motif representation learning, contrastive learning

## Abstract

Accurate molecular property prediction underpins progress in drug discovery, medicinal chemistry, and molecular design and depends critically on the quality of the learned molecular representations. While Graph Neural Networks (GNNs) and Graph Transformers have advanced this field considerably, most existing approaches emphasize a single structural perspective and struggle to jointly capture local connectivity, higher-order substructures, explicit topology, and long-range dependencies in a single learning process. This work introduces Topology-Guided Multi-Level Graph Learning (TMGL), a unified framework that integrates topology-enhanced node encoding, motif-guided representation learning, a Multi-Scale Graph Isomorphism Network, topology-aware graph transformer attention, adaptive multi-scale feature fusion, and cross-level contrastive learning into a single end-to-end architecture. By coupling topology preservation and motif consistency objectives with hierarchical contrastive alignment across node-, motif-, and graph-level embeddings, TMGL enables these complementary structural views to interact directly rather than being combined post hoc. The framework was evaluated on seven MoleculeNet benchmark datasets spanning binary classification, multi-label classification, and regression tasks. TMGL achieved Receiver Operating Characteristic Area Under the Curve (ROC-AUC) scores of 0.937, 0.960, 0.962, and 0.723 on BACE, BBBP, ClinTox, and SIDER, respectively, and RMSE values of 0.611, 1.293, and 0.436 on ESOL, FreeSolv, and Lipophilicity, outperforming a broad set of state-of-the-art baselines on most classification benchmarks and on Lipophilicity, while remaining competitive on ESOL and FreeSolv. Ablation experiments confirmed that each architectural component contributes meaningfully to overall performance, and embedding visualizations further illustrated the discriminative, class-consistent structure of the learned representation space. These results demonstrate the value of jointly modeling complementary structural perspectives for molecular graph learning and highlight the potential of the TMGL framework for applications in computational chemistry and drug discovery.

## 1. Introduction

Graph-structured data are ubiquitous in computational chemistry, bioinformatics, social network analysis, molecular property prediction, and drug discovery [1,2]. In molecular applications, atoms and chemical bonds can be naturally represented as nodes and edges, making graph representation learning an effective approach for modeling complex molecular structures [3,4]. For tasks such as toxicity prediction [5], solubility estimation, and biological activity analysis [6], where predictive accuracy depends heavily on how well structural and semantic information is learned from molecular graphs, this ability is particularly important [7,8].

Recent advances in deep learning have significantly improved graph representation learning by enabling models to learn directly from graph structures without relying on handcrafted descriptors [9]. Graph neural networks (GNNs) have emerged as one of the leading approaches for learning from graph-structured data [10]. Representative architectures such as Graph Convolutional Networks (GCNs), Graph Attention Networks (GATs), and Graph Isomorphism Networks (GINs) repeatedly aggregate information from neighboring nodes to produce rich node and graph representations [11,12]. These models have demonstrated high predictive accuracy across a wide range of molecular prediction problems by capturing local structural features and leveraging connectivity information in molecular graphs [13].

Despite their success, most GNN-based architectures are rooted in local message-passing operations. Information is diffused by repeatedly aggregating node features, yielding learned representations that are primarily based on local graph neighborhoods. This local aggregation becomes problematic as graph complexity increases, making it impossible to model interactions between remote nodes and capture global structural dependencies. In addition, deeper message-passing networks tend to introduce oversmoothing and oversquashing, which may diminish the discriminative power of node representations and limit the amount of information that can flow through the graph [14].

Recently, graph transformers have attracted considerable interest because of these drawbacks. Transformer-based models use self-attention mechanisms to enable direct connections between any two nodes in a graph, thereby capturing long-range context. The attention mechanism can be further enhanced by incorporating a structural encoding that provides additional information about the underlying graph topology [15]. This gives graph transformers an advantage in modeling global dependencies that are not easily captured by standard neighborhood aggregation [16].

However, several challenges remain in molecular graph representation learning. First, most previous methods rely primarily on either local message passing or global attention and cannot fully leverage complementary structural information for representation learning [17]. Second, higher-order semantic information, while shown to be useful, is typically added on top of the graph rather than integrated into its topology and long-range structural dependencies, so different levels of molecular information interact only weakly [18]. Third, topology-aware structural descriptors are usually treated as extra node features rather than built into the representation learning process itself [19]. As a result, local neighborhood information, higher-order structural semantics, explicit topological features, and global graph dependencies tend to be learned in isolation rather than as part of a unified framework.

Topology-aware learning, motif representation learning, graph transformers, and contrastive learning have each been studied extensively on their own, but their combination has been explored only partially [20,21]. Getting these structural perspectives to interact throughout the representation learning process, rather than remaining side by side, remains largely unexplored in molecular property prediction. This work proposes the Topology-Guided Multi-Level Graph Learning (TMGL) framework, which jointly combines topology-aware structural encoding, motif-guided semantic learning, Multi-Scale GIN-based local feature extraction, topology-aware graph transformer attention, adaptive multi-scale feature fusion, and hierarchical contrastive learning within a single end-to-end architecture. Rather than treating these as separate modules, the framework allows local connectivity, higher-order structural semantics, explicit topological information, and long-range graph dependencies to interact directly, producing richer molecular representations [22].

The proposed framework first augments each molecular graph with topology-aware structural descriptors that complement the original atomic features by explicitly characterizing each node’s structural role. Chemically meaningful motifs are then extracted to capture higher-order structural patterns that neighborhood aggregation alone cannot adequately represent. A Multi-Scale GIN encoder processes the enriched node representations to learn expressive local structural features, which are then refined by a topology-aware graph transformer that captures long-range structural dependencies through topology-guided self-attention. A hierarchical contrastive learning strategy aligns node-level, motif-level, and graph-level representations to improve consistency across structural scales. In addition, topology preservation and motif consistency objectives help maintain structural fidelity during optimization. At the same time, adaptive multi-scale feature fusion and attention-based graph pooling integrate complementary local, motif-level, and global representations into a unified graph embedding for downstream molecular property prediction. Through the collaborative interaction of these complementary structural perspectives, the proposed framework learns richer, more discriminative molecular representations than approaches that rely primarily on a single representation-learning strategy.

This work presents a unified graph representation learning framework that integrates topology-aware structural encoding, motif-guided semantic learning, transformer-based global reasoning, adaptive multi-scale feature fusion, and hierarchical contrastive learning within a single end-to-end optimization framework for molecular property prediction. By encouraging collaboration among local, motif-level, and global representations, the proposed framework effectively exploits complementary structural information rather than learning each representation independently. Extensive experiments on seven molecular property prediction benchmark datasets, together with visualization analyses and ablation studies, demonstrate that the proposed approach consistently improves predictive performance and robustness across both classification and regression tasks.

## 2. Related Work

Graph representation learning has emerged as a core research area in machine learning and has found wide applications in molecular property prediction, drug discovery, recommender systems, social network analysis, and knowledge graph reasoning. Over the last decade, similarity measures have evolved from manually designed methods to deep neural network architectures that automatically learn expressive graph embeddings. The literature encompasses several broad lines of research, including graph kernels, graph neural networks, graph transformers, motif-aware learning, contrastive representation learning, and topology-aware modeling [23].

The earliest studies relied on graph kernels, which measure the similarity between two graphs by comparing predefined graph structures such as shortest paths, random walks, graphlets, or subtrees. Representative methods, such as the Shortest-Path Kernel [24] and the Weisfeiler–Lehman (WL) Kernel [25], laid the groundwork for graph comparison and performed competitively on a set of benchmark tasks. Although they are interpretable, they depend heavily on manually designed similarity functions and incur high computational cost during implementation, limiting their scalability and ease of application to complex graph domains.

The development of GNNs revolutionized graph representation learning by providing an end-to-end solution for extracting features from a graph’s topology. This was achieved with GCNs, which use local messages to share information with other vertices within a local neighborhood [26], and with later variants that scale the approach by sampling neighborhoods or using more efficient aggregation methods [27]. These architectures are highly successful for many graph learning problems. However, they model only local interactions, and as the network becomes deeper, their ability to capture long-range interactions is constrained, and the discriminative power of node representations gradually diminishes due to oversmoothing.

To improve the expressiveness of neighborhood aggregation, attention mechanisms were incorporated into graph learning via GATs, in which adaptive attention weights enable the model to focus more on informative neighboring nodes during feature aggregation [28]. GINs further enhanced representational ability by introducing injective aggregation functions with discriminative power comparable to the Weisfeiler–Lehman graph isomorphism test [29]. These architectures are good at retaining local features, but they still rely on neighborhood aggregation for computation, limiting their ability to model global structural dependencies. Graph transformers were introduced, in part, to address this limitation, shifting graph representation learning toward global self-attention and enabling each node to interact directly with every other node in the graph [30]. A variety of transformer-based networks incorporate positional encodings based on shortest-path distances, Laplacian eigenvectors, centrality metrics, and other structural descriptors to preserve structural awareness [31]. These additions significantly enhance the models’ ability to capture global graph semantics and long-range dependencies. However, these models are largely based on pairwise node interactions; higher-order, chemically meaningful structures, such as functional groups and molecular motifs, are represented only implicitly.

Because higher-order structural organization matters, motif-aware learning has become a useful approach for capturing chemically meaningful substructures [32]. Early work incorporated graphlets, cycles, cliques, and functional groups to extend graph representations beyond individual edges [33]. Hierarchical molecular graph self-supervised learning (HiMol) later showed that learning hierarchical representations via self-supervised objectives markedly improves the quality of molecular representations [2]. The Atom-motif contrastive transformer (AMCT) combines motif-aware attention with motif-level contrastive learning within a graph transformer, demonstrating that explicitly modeling motif-level semantics yields more informative molecular graph representations [34]. Together, these studies establish chemically meaningful motifs as a key component of molecular representation learning and position motif-aware learning as a promising research direction [35].

Recent advances in graph-based deep learning for structural bioinformatics further demonstrate the value of combining complementary structural representations. Structural features of the RNA backbone can be obtained by GCNfold, which combines graph convolutional networks with Transformer-based sequence, structure fusion, and captures long-range dependencies in RNA secondary structure prediction [36]. PaxNet is a multiplex graph neural network that separately models local and non-local interactions, and incorporates attention-based fusion to combine various types of interactions for 3D structure assessment of RNA [37]. In the same way, to accurately account for the topological features of RNA secondary structures, in addition to sequence information, RNASSR-Net resolves the problems of RNA–protein binding prediction by explicitly modelling the topological properties of the secondary structures of RNA [38]. More recently, GraphaRNA has been used to represent RNA as graphs and to leverage a diffusion-based generative model with graph neural networks to model interactions between atoms and local structural descriptors for 3D structure prediction of RNA [39]. Together these studies demonstrate the need for a multi-modal approach to modeling local, non-local, topological and higher-order molecular or biological structure, including graph-based structural information. These developments further encourage the need for a unified representation learning framework for graphs that can collectively leverage multiple structural aspects for the prediction of molecular properties.

Another important area is graph contrastive learning, which learns discriminative graph representations by pulling together different views of a graph and pushing apart views of unrelated graphs [40]. Previous studies have used contrastive objectives at the node [41], subgraph level [42], and graph level [43], achieving good generalization performance, particularly when labeled data is limited [44]. Recently, hierarchical contrastive learning has been applied to align multiple levels of semantic representations to enhance consistency and robustness [45,46]. However, these contrastive objectives are typically optimized without incorporating topology-aware structural information and motif-guided semantic representations. In fact, interactions among local structural features, higher-order motifs, global information about graph dependencies, and topology-aware information remain only partially explored.

Alongside these developments, topology-aware graph learning has incorporated explicit structural descriptors, such as node degree, clustering coefficient, and other graph-theoretic measures, to enrich graph representations and improve downstream prediction performance [47,48]. These descriptors provide valuable information about the structural roles of nodes and have proven effective across a variety of graph learning tasks. Nevertheless, topology-aware information is commonly incorporated as auxiliary node attributes or positional encodings rather than integrated throughout the entire representation learning process. As a result, topology-aware features, motif representations, global graph semantics, and contrastive objectives are generally optimized separately rather than collaboratively contributing to graph representation learning [49].

Recent molecular graph learning models have increasingly integrated complementary ideas. For example, HiMol improves molecular representations through hierarchical self-supervised learning across multiple structural levels [2]. In contrast, AMCT combines motif-aware attention with motif-level contrastive learning within a graph transformer architecture [34]. Although these methods demonstrate the effectiveness of hierarchical learning and motif-guided representations, they primarily focus on specific aspects of molecular graph learning rather than enabling topology-aware structural encoding, local feature learning, higher-order motif reasoning, global transformer attention, structural consistency, adaptive multi-scale representation fusion, and cross-level representation alignment to interact within a unified optimization framework. Consequently, the collaborative learning of these complementary structural perspectives remains relatively underexplored. This observation motivates the TMGL framework proposed in this work, in which topology-aware feature extraction, motif representation learning, Multi-Scale GIN encoding, topology-aware graph transformer attention, topology preservation, motif consistency, adaptive multi-scale feature fusion, attention-based graph pooling, and hierarchical contrastive learning are jointly optimized to learn richer and more discriminative molecular graph representations.

## 3. Results and Discussions

### 3.1. Performance and Representation Analysis

The predictive performance of the proposed TMGL framework was compared against a diverse set of representative molecular graph learning methods, spanning traditional graph neural networks, transformer-based architectures, motif-aware models, self-supervised approaches, and recent large-scale molecular representation learning frameworks. The comparison covers SchNet [50], graph convolution (GraphConv) [51], directed message passing neural network (DMPNN) [4], molecular pre-training graph (MPG) [52], knowledge graph-enhanced molecular contrastive learning with functional prompt (KANO) [53], HiMol [2], automated machine learning (AutoML) [54], graph representation from self-supervised message passing transformer (GROVER) [17], molecular language transformer (MolFormer) [55], motif-based graph self-supervised learning (MGSSL) [56], prototype-guided multimodal framework (ProtoMol) [57], molecular graph-based extended long short-term memory framework (MolGraph-xLSTM) [58], and atom-motif contrastive transformer (AMCT) [34], which together represent the current state of the art in molecular property prediction and provide a comprehensive benchmark for the proposed framework. Results are summarized in Table 1, with classification performance measured by ROC-AUC and regression performance by RMSE.

TMGL performs competitively or better across both classification and regression benchmarks. On BACE, it achieves an AUC-ROC of 0.937±0.006, outperforming all competing methods and effectively identifying β-secretase inhibitors. On BBBP, the framework achieves an AUC-ROC of 0.960±0.016, again surpassing prior state-of-the-art methods; the learned molecular representations appear to capture structural characteristics associated with blood–brain barrier permeability. On ClinTox, TMGL reaches the highest AUC-ROC of 0.962±0.014, consistent with its ability to model toxicity-related molecular structures. On the more challenging multi-label SIDER benchmark, it achieves an average ROC-AUC of 0.723±0.012, outperforming all compared methods. Given that SIDER spans 27 independent toxicity prediction tasks with pronounced class imbalance, this result indicates that the representations are robust and generalize across multiple adverse drug reaction tasks. The regression results follow the same pattern. On the Lipophilicity dataset, TMGL attains an RMSE of 0.436±0.006, indicating accurate prediction of the octanol–water partition coefficient. TMGL achieves an RMSE of 0.611±0.050 on ESOL, demonstrating its ability to accurately predict aqueous solubility, with performance comparable to that of MolGraph-xLSTM. On FreeSolv, it achieves an RMSE of 1.293±0.071, outperforming most conventional GNN-based methods and being competitive with several recent large-scale pretrained models. Together, these results indicate good generalization across diverse physicochemical property prediction tasks.

The improvements in both classification and regression tasks seem to stem from a combination of complementary representation learning components. Topology-aware structural descriptors include structural information represented as a graph, appended to the original atomic features, and motif representation learning enriches this structural information by recovering chemically meaningful higher-order substructures. The multi-level contrastive learning objective increases the consistency of node-level, motif-level, and graph-level representations, while the topology preservation loss and the motif consistency loss drive the embedding space toward structural coherence. The learned feature fusion module dynamically balances the weights of local structural information, motif-aware semantic representations, and global graph representations, adaptively assigning weights to the most informative features when building a model for a molecular graph. Together, these components enable TMGL to learn richer molecular representations than those learned by a representation learning strategy primarily based on a single representation.

The training time per epoch was measured on representative molecular property prediction datasets to assess the computational efficiency of the proposed TMGL framework. All of the experiments were performed in a single computational setup comprising an NVIDIA Tesla T4 GPU with CUDA support. Figure 1 shows the training time taken per epoch by BBBP, ClinTox, FreeSolv and ESOL after 300 epochs. The results demonstrate fairly consistent training-time profiles from epoch to epoch, with the differences mainly due to the size of the datasets and the architecture of the graphs. These are empirical measures of the computational cost of TMGL at training time. The training-time variation observed also suggests that the computational cost is not significantly increased with training progress, but rather stays reasonably low. Such measurements are useful and practical in the sense that they offer information on the costs of the proposed multi-level architecture, which is integrated in TMGL, with topology features, motif representations, multi-scale GIN encoding, global graph modeling and attention-based representation learning. A direct runtime comparison with baseline models was not performed because their reported results were obtained under heterogeneous hardware and implementation environments. In addition, inference latency and stage-wise preprocessing costs were not independently profiled in the present study. Standardized end-to-end computational benchmarking under identical experimental conditions will therefore be considered in future work.

Other classification sets were used to assess the framework’s discriminative power, and ROC curves were plotted for the four sets (BACE, BBBP, ClinTox, and SIDER) and are shown in Figure 2. The ROC curve shows the relationship between the true positive rate and the false positive rate across all possible thresholds and can serve as a threshold-independent measure of molecular classification. TMGL’s ROC curves are close to the upper-left corner across all datasets, consistent with strong discriminative performance. ClinTox demonstrates the best discriminatory power, followed by BBBP and BACE. Although SIDER is a multi-label dataset, its performance remains reasonable but is clearly affected by severe class imbalance. These observations are consistent with the quantitative ROC-AUC results in Table 1.

To better explore the learned molecular representations, three of the most popular dimensionality reduction methods were used to compare the graph embeddings obtained from TMGL: Uniform Manifold Approximation and Projection (UMAP), Principal Component Analysis (PCA), and t-distributed Stochastic Neighbor Embedding (t-SNE). Typical results for BACE and BBBP are shown in Figure 3. In all three techniques, molecules of the same class cluster together, while molecules from different classes remain well separated. The representations generated by TMGL thus seem to retain structural similarity and class-specific semantics in the representation space, which explains its excellent predictive performance across all molecular property prediction tasks tested.

Scatter plots were also used to evaluate the regression performance of TMGL on the FreeSolv, ESOL, and Lipophilicity datasets by comparing the model-predicted values with the ground-truth values, as illustrated in Figure 4. The samples cluster around the diagonal reference line, indicating strong agreement between the predicted and measured values. Across all three datasets, the predicted and measured values show good agreement, with most data points distributed close to the ideal-prediction line. The structure is particularly well correlated along the diagonal in ESOL and Lipophilicity, which aligns with the idea that the structure reflects the physicochemical relationships between molecular solubility and lipophilicity. The scatter is slightly larger for FreeSolv, probably because there are fewer structures and because the free energy of hydration is more difficult to predict. Despite this, most of the samples are near the ideal-prediction line, and the regression remains stable even on this more difficult dataset.

Residual analysis was conducted to systematically investigate prediction errors for the FreeSolv, ESOL, and Lipophilicity datasets, with residuals defined as the difference between the actual and predicted values, as shown in Figure 5. A good regression model should yield residuals that are randomly scattered around a mean of zero, with no clear systematic pattern. The residuals for FreeSolv, ESOL, and Lipophilicity cluster around zero, suggesting no significant prediction bias. The residual ranges for ESOL and Lipophilicity are relatively narrow, indicating stable, consistent predictions across molecular samples. The FreeSolv dataset exhibits a wider residual range, consistent with the greater complexity of predicting hydration free energy and the smaller number of training examples.

Residual histograms were then examined to further assess the distribution of prediction errors, as shown in Figure 6. For well-calibrated regression models, residual distributions should be centered at 0 and approximately symmetric. The residual distributions for FreeSolv, ESOL, and Lipophilicity are centered around zero and approximately bell-shaped. The residual ranges are narrower for ESOL and Lipophilicity, suggesting that prediction errors are generally small. The FreeSolv distribution is marginally broader, as shown in the residual analysis, and is indicative of the increased variance for this data set.

The experimental results clearly demonstrate that TMGL is effective on both classification and regression benchmarks. The framework performs best in terms of AUC-ROC on BACE, BBBP, and ClinTox, and achieves low RMSE on FreeSolv, ESOL, and Lipophilicity. These results suggest that the proposed architecture generalizes well across various molecular property prediction tasks.

These performance gains stem from several architectural innovations. The topology feature extraction module enriches node representations with structural features before passing them to the message passing module, thereby encoding key graph properties. The motif extraction module adds a second level of structural semantics that traditional edge aggregation methods often ignore. The Multi-Scale GIN encoder models local neighborhood interactions, and the topology-aware graph transformer uses topology-guided self-attention to capture long-range dependencies. Moreover, the cross-level contrastive learning strategy ensures consistency across node-, motif-, and graph-level representations, yielding more discriminative molecular embeddings and improved predictive performance across various chemical property prediction tasks.

### 3.2. Ablation Analysis

The complete TMGL framework consistently achieves the best overall performance across all five benchmark datasets, as shown in Table 2. It reports ROC-AUC scores of 0.937±0.006 on BACE, 0.962±0.014 on ClinTox, and 0.723±0.012 on SIDER, and the lowest RMSE values of 0.611±0.050 on ESOL and 1.293±0.071 on FreeSolv. These results indicate that combining topology-aware structural information, motif-aware semantic representations, local graph representations, and complementary auxiliary objectives enables TMGL to produce more informative molecular representations for both classification and regression tasks.

Removing any individual component reduces performance relative to the full model, as shown in Table 2. Excluding the topology feature extraction module noticeably degrades performance across all datasets, with a particularly large increase in FreeSolv RMSE from 1.293±0.071 to 2.198±0.026; explicit graph-theoretic descriptors provide structural information beyond the original atomic attributes. Removing motif representation learning likewise consistently lowers predictive performance, particularly reducing the BACE ROC-AUC from 0.937±0.006 to 0.894±0.008, confirming that chemically meaningful motifs capture higher-order molecular semantics that local neighborhood aggregation alone cannot represent. The contribution of the proposed optimization objectives is also evident in the ablation results. The removal of multi-level contrastive learning reduces predictive performance on both classification and regression datasets, yielding the lowest SIDER ROC-AUC of 0.622±0.071 and the highest ESOL RMSE of 0.748±0.017. Aligning representations across structural levels seems to enhance the discriminative power of the learned representations.

Similarly, eliminating the topology preservation loss deprives the model of its ability to preserve structural relationships between nodes in the embedding space and substantially decreases the ClinTox ROC-AUC to 0.892±0.007. Removing the motif consistency loss reduces the coherence of motif representations and, in turn, degrades prediction accuracy, with a particularly large reduction in BACE ROC-AUC to 0.891±0.005. Replacing the learned feature fusion mechanism also yields lower performance than the full framework, confirming that adaptive integration of local, motif-level, and global representations contributes to the overall effectiveness of TMGL. Overall, the ablation study demonstrates that TMGL’s success does not depend on any single architectural component; rather, it arises from the combined contributions of topology feature extraction, motif representation learning, multi-level contrastive learning, topology preservation, motif consistency, and learned feature fusion. Together, these components enrich the learned molecular representations and deliver consistent performance improvements across both classification and regression datasets.

## 4. Methodology

Graph representation learning aims to learn informative graph-level embeddings that effectively support a wide range of downstream tasks, including molecular classification, toxicity prediction, molecular property estimation, and graph classification. Although existing GNN models have achieved remarkable success in graph learning, many approaches rely primarily on local neighborhood aggregation and often fail to capture higher-order structural patterns and long-range dependencies inherent in graph-structured data. Furthermore, graph topology information, which provides valuable structural insights, is often underutilized during representation learning. To address these limitations, this work proposes a novel TMGL framework that jointly integrates topology-enhanced learning, motif-guided representation learning, and local structural encoding within a unified representation learning pipeline, as illustrated in Figure 7.

The architecture of the proposed TMGL framework is illustrated in Figure 8. The framework consists of topology feature extraction, motif extraction, Multi-Scale GIN encoding, global graph transformer representation, motif representation learning, cross-level contrastive learning, topology preservation, motif consistency learning, adaptive feature fusion, attention-based graph pooling, and graph-level prediction, which are described in this section. Given an input graph G=(V,E,X), where *V*, *E*, and *X* denote the node set, edge set, and node feature matrix, respectively, the framework first extracts topology-enhanced descriptors to enrich the original node features with structural information. A motif extraction module then identifies higher-order structural patterns that provide complementary graph semantics. These graph representations are then passed through a Multi-Scale GIN encoder to capture local structural dependencies. Motif-level representations and contrastive learning objectives are then used further to improve the quality of the learned graph embeddings.

### 4.1. Topology Feature Extraction

Node attributes alone rarely capture the structural role a node plays in a graph. Graph-theoretic measures provide complementary information about local connectivity, neighborhood organization, and node importance. TMGL therefore augments the original node features with topology-enhanced descriptors before graph encoding, as illustrated in Figure 9.

For each node, three complementary topological descriptors are computed: degree centrality, clustering coefficient, and betweenness centrality. These descriptors are represented as:

(1) $$t_{i}=\left[d_{i},c_{i},b_{i}\right]$$

where $d_{i}$, $c_{i}$, and $b_{i}$ denote the degree centrality, clustering coefficient, and betweenness centrality of node *i*, respectively. The topology descriptor is concatenated with the original node feature vector to produce a topology-enhanced representation:

(2) $$x_{i}^{topo}=\left[x_{i}∥t_{i}\right]$$

where ‖ denotes the concatenation operator. The topology-enhanced feature matrix is therefore:

(3) $$X^{topo}=\left[x_{1}^{topo},x_{2}^{topo},\ldots ,x_{N}^{topo}\right]$$

where *N* denotes the total number of nodes in the molecular graph. The topology-enhanced feature matrix serves as input to the subsequent motif extraction and graph encoding modules. By building graph topology directly into node representations, the framework retains both attribute information and structural characteristics, providing downstream learning with a richer representation.

### 4.2. Motif Extraction Module

Topology-enhanced node features capture important structural features but fail to capture the higher-order structure of the graph. Substructures such as rings and functional groups are important for determining molecular properties and are common in molecular graphs. In this context, TMGL introduces a motif extraction module to capture higher-order structural semantics, as shown in Figure 8. Given the input graph, the motif extraction procedure identifies a collection of recurring structural patterns:

(4) $$M=\{M_{1},M_{2},\ldots ,M_{K}\}$$

where *K* denotes the total number of motifs extracted from the molecular graph, and each motif represents a subset of graph nodes participating in a specific higher-order substructure. To encode node-to-motif relationships, a motif incidence matrix is defined as:

(5) $$M\in R^{N\times m}$$

where *N* denotes the number of nodes and *m* represents the number of extracted motifs. Each matrix element indicates whether a node belongs to a particular motif. These extracted motifs provide structural information that complements the topology-enhanced node features. Unlike conventional message passing, which models only pairwise interactions, motif representations capture higher-order structural dependencies that local neighborhood aggregation alone struggles to learn. The motif assignments are then carried over to the next steps of the framework (as shown in Figure 8) to learn motif representations and hierarchically contrast them.

### 4.3. Multi-Scale GIN Encoder

After topology enhancement and motif extraction, the graph is encoded using a Multi-Scale GIN to learn expressive local node representations, as illustrated in Figure 8. GIN is chosen for its strong discriminative power and its theoretical link to the Weisfeiler–Lehman graph isomorphism test. Through iterative neighborhood aggregation, the encoder captures local structural patterns while gradually expanding its receptive field. Given the topology-enhanced feature matrix $X^{topo}$, node representations are updated through multiple GIN layers according to:

(6) $$h_{i}^{\left(k\right)}={\mathrm{MLP}}^{\left(k\right)}\left(\left(1+\epsilon^{\left(k\right)}\right)h_{i}^{\left(k-1\right)}+\sum_{j\in N(i)}h_{j}^{\left(k-1\right)}\right)$$

where $h_{i}^{\left(k\right)}$ denotes the representation of node *i* at the *k*-th layer, N(i) is the neighborhood of node *i*, ${\mathrm{MLP}}^{\left(k\right)}$ represents a learnable multilayer perceptron, and $\epsilon^{\left(k\right)}$ is a trainable parameter that controls the central node’s contribution during aggregation. Stacking multiple GIN layers enables information to propagate from progressively larger neighborhoods, allowing the encoder to learn hierarchical local representations while preserving discriminative structural information. The resulting local node embeddings are represented as:

(7) $$H_{local}=\left\{h_{1},h_{2},\ldots ,h_{N}\right\}$$

where $H_{local}$ denotes the set of node representations learned via multi-scale neighborhood aggregation.

### 4.4. Graph Transformer for Global Representation Learning

Although the Multi-Scale GIN encoder effectively captures local neighborhood information, message passing alone has limited capacity to model interactions between distant nodes. To address this limitation, TMGL incorporates a graph transformer layer, as shown in Figure 8, to aggregate global information via multi-head self-attention and integrate structural information from the graph topology. Given the local node representations $H_{local}$, linear projections are first applied to generate the query (*Q*), key (*K*), and value matrices (*V*):

(8) $$Q=H_{local}W_{Q},\;K=H_{local}W_{K},\;V=H_{local}W_{V}$$

where $W_{Q}$, $W_{K}$, and $W_{V}$ are learnable projection matrices. The attention scores are computed using scaled dot-product attention:

(9) $$A=\frac{QK^{T}}{\sqrt{d}}$$

where *T* denotes the matrix transpose, and *d* the dimensionality of each attention head. To incorporate structural information into the attention mechanism, the shortest-path distance between node pairs is computed from the graph’s connectivity. The resulting shortest-path matrix is transformed into an attention bias matrix *B*, in which reachable node pairs receive distance-based penalties and unreachable pairs are assigned a large negative value. The attention scores are then updated as:

(10) $$A^{\prime }=A+B$$

The normalized attention weights are obtained by applying the Softmax function, and the updated node representations are computed as:

(11) $$H_{global}=\mathrm{Softmax}\left(A^{\prime }\right)V$$

Finally, residual connections, layer normalization, and a feed-forward network are used to refine node representations and improve training stability. The resulting global representations complement the local structural features learned by the Multi-Scale GIN encoder, providing additional contextual information for the subsequent motif learning and adaptive feature fusion modules.

### 4.5. Motif Representation Learning

In addition to node-level representations, TMGL learns motif-level representations to preserve higher-order structural characteristics of molecular graphs. Because motifs consist of groups of structurally related nodes, aggregating node embeddings within each motif yields a compact representation of chemically meaningful substructures. Figure 10 provides an illustrative example of chemical motif extraction from an atom-level molecular graph. It demonstrates how meaningful substructures (as functional groups) can be identified and represented as motif-level units.

Let $M_{k}$ denote the set of nodes in the *k*-th motif. The corresponding motif representation is computed by averaging the node embeddings within the motif:

(12) $$h_{k}^{motif}=\frac{1}{|M_{k}|}\sum_{i\in M_{k}}h_{i}$$

where $h_{i}$ denotes the learned node embedding and $|M_{k}|$ represents the number of nodes contained in the motif $M_{k}$. This aggregation preserves the overall structural characteristics of each motif while reducing the influence of individual node variations. The collection of motif representations is expressed as:

(13) $$H_{motif}=\left\{h_{1}^{motif},h_{2}^{motif},\ldots ,h_{K}^{motif}\right\}$$

where *K* denotes the total number of extracted motifs. These motif embeddings provide complementary structural information that is subsequently utilized alongside node-level representations during contrastive learning and feature fusion.

### 4.6. Cross-Level Contrastive Learning

TMGL uses a cross-level contrastive learning objective, as shown in Figure 8, to promote consistency across structural levels. The goal is to align the node-level, motif-level, and graph-level representations while enhancing the discriminative capacity of the learned embedding space [59]. Given a pair of positive representations $\left(z_{i},z_{j}\right)$, the contrastive objective is formulated using the InfoNCE loss:

(14) $$L_{InfoNCE}=-\log\frac{\exp(\mathrm{sim}\left(z_{i},z_{j}\right)/\tau )}{\sum_{k}\exp\left(\mathrm{sim}\left(z_{i},z_{k}\right)/\tau \right)}$$

where sim(·,·) denotes cosine similarity and τ is the temperature parameter controlling the distribution of similarities. The overall cross-level contrastive objective combines interactions between node-level, motif-level, and graph-level representations:

(15) $$L_{C}=L_{NM}+L_{MG}+L_{NG}$$

where $L_{NM}$, $L_{MG}$, and $L_{NG}$ represent the node–motif, motif–graph, and node–graph contrastive losses, respectively. By jointly optimizing these objectives, the framework encourages representations across different structural levels to remain semantically consistent while preserving both local and global structural information for molecular property prediction.

### 4.7. Topology Preservation Module

Although the Multi-Scale GIN encoder and the graph transformer learn expressive node representations, preserving the intrinsic graph topology within the learned embedding space is essential for maintaining structural consistency. To achieve this objective, TMGL incorporates a topology preservation module, as illustrated in Figure 8, that encourages neighboring nodes in the molecular graph to remain close in the embedding space while separating nodes that are not directly connected. Let $H=\{h_{1},h_{2},\ldots ,h_{N}\}$ denote the learned node embeddings. Each embedding is first normalized, and the pairwise cosine distance between node representations is computed as:

(16) $$D_{ij}=1-\frac{h_{i}^{T}h_{j}}{∥h_{i}∥∥h_{j}∥}$$

where $D_{ij}$ represents the embedding distance between nodes *i* and *j*. Using the graph adjacency matrix *A*, the topology preservation objective consists of two complementary components. The first component minimizes the distances between connected node pairs:

(17) $$L_{pos}=\frac{1}{\left|E\right|}\sum_{(i,j)\in E}D_{ij}$$

where *E* denotes the set of graph edges. The second component encourages disconnected node pairs to remain sufficiently separated by using a margin-based constraint:

(18) $$L_{neg}=\frac{1}{|\bar{E}|}\sum_{(i,j)\notin E}\max\left(0,\delta -D_{ij}\right)$$

where δ is the margin parameter and $\bar{E}$ denotes the set of unconnected node pairs. The overall topology preservation loss is therefore expressed as:

(19) $$L_{Topo}=L_{pos}+L_{neg}$$

Jointly minimizing these two objectives enables the learned node representations to preserve the structural connectivity of the original molecular graph while improving the discriminative quality of the embedding space.

### 4.8. Motif Consistency Module

TMGL also incorporates a motif consistency module, as shown in Figure 8, to encourage nodes belonging to the same motif to learn similar embedding representations and preserve higher-order structural information. Chemically meaningful motifs represent recurring substructures of molecules; enforcing consistency for each motif will provide greater robustness for each learned motif and better preserve higher-order structural information [60]. The node embeddings associated with the motif are first clustered by motif membership, using the node–motif membership matrix. The structural consistency of each motif is quantified as the cosine similarity between node embeddings and is calculated for each motif with more than one node. The objective of motif consistency is described as:

(20) $$L_{Motif}=\frac{1}{K}\sum_{k=1}^{K}\left(1-\frac{1}{|P_{k}|}\sum_{\left(i,j\right)\in P_{k}}\mathrm{sim}\left(h_{i},h_{j}\right)\right)$$

where *K* denotes the total number of extracted motifs, $P_{k}$ represents the set of node pairs belonging to the *k*-th motif, $h_{i}$ and $h_{j}$ denote the learned node embeddings, and sim(·,·) denotes the cosine similarity function. The loss minimizes the embedding dissimilarity among nodes within the same motif, thereby encouraging structurally related nodes to occupy nearby positions in the embedding space. By enforcing representation consistency within each motif, the proposed framework learns more discriminative motif-level structural patterns while preserving the intrinsic relationships among nodes that participate in the same molecular substructure. This complementary objective further enhances the quality of the learned graph representations for molecular property prediction.

### 4.9. Adaptive Feature Fusion

The proposed TMGL framework learns complementary representations from local neighborhood aggregation, motif-level structural information, and global contextual modeling. To integrate these heterogeneous features effectively, TMGL employs an adaptive feature fusion module, as illustrated in Figure 8. Let $H_{local}$, $H_{motif}$, and $H_{global}$ denote the graph-level representations obtained from the Multi-Scale GIN encoder, motif representation module, and graph transformer, respectively. Instead of assigning fixed contributions to these representations, TMGL learns three trainable fusion coefficients that are normalized using the Softmax function:

(21) [α,β,γ]=Softmax(w)

where *w* denotes the learnable fusion parameters satisfying:

(22) α+β+γ=1

The final graph representation is obtained via a weighted linear combination of the three complementary representations:

(23) $$H_{F}=\alpha H_{local}+\beta H_{motif}+\gamma H_{global}$$

By learning the fusion weights jointly with the network parameters, the framework automatically determines the relative importance of local structural information, motif semantics, and global contextual representations for molecular property prediction.

### 4.10. Attention-Based Graph Pooling

Following the extraction of local node representations, a graph-level representation is constructed by aggregating node embeddings using an attention-based pooling mechanism, as illustrated in Figure 8. Unlike conventional pooling strategies that assign equal importance to all nodes, the proposed attention pooling module learns adaptive importance scores for individual nodes, enabling the model to focus on structurally informative regions of the molecular graph [61]. Given the node representations $H=\{h_{1},h_{2},\ldots ,h_{N}\}$, an attention score is first computed for each node through a learnable linear transformation followed by a sigmoid activation. The resulting scores are normalized using the Softmax function to obtain attention weights:

(24) $$a_{i}=\frac{\exp(s_{i})}{\sum_{j=1}^{N}\exp\left(s_{j}\right)}$$

where $s_{i}$ denotes the attention score for node *i*. The graph-level representation is then computed as the weighted aggregation of node embeddings:

(25) $$h_{G}=\sum_{i=1}^{N}a_{i}h_{i}$$

The attention-based pooling mechanism enables the framework to emphasize structurally significant nodes while suppressing less informative ones, yielding a compact, discriminative graph-level representation. This graph representation then passes through a projection head and feeds into the cross-level contrastive learning module. At the same time, the final molecular property prediction relies on the adaptively fused graph representation produced by the adaptive feature fusion module.

### 4.11. Prediction Layer

The adaptive feature fusion module builds a unified graph-level representation by combining local structural information, motif-aware semantic features, and global topology-aware representations. The adaptively fused graph-level representation is passed to a task-specific prediction layer, as illustrated in Figure 8, to perform molecular property prediction. For classification tasks, the prediction head consists of a three-layer MLP with two fully connected hidden layers, each followed by a Rectified Linear Unit (ReLU) activation and dropout regularization [62], and a final linear output layer [63]. For binary classification datasets, the output layer contains two neurons corresponding to the molecular classes, and the model is optimized using the Cross-Entropy loss. For multi-label classification datasets, such as Side Effect Resource (SIDER), the output layer contains one independent neuron for each prediction task. The output logits are transformed using the Sigmoid activation function, and each label is optimized independently using the Binary Cross-Entropy with Logits (BCEWithLogits) loss. For regression tasks, the same MLP architecture is employed, except that the final output layer contains a single neuron that directly predicts a continuous molecular property value without applying any output activation function.

To improve the discriminative capability and structural consistency of the learned molecular representations, the prediction network is jointly optimized with the proposed cross-level contrastive learning, topology preservation, and motif consistency objectives. Accordingly, the overall optimization objective differs for classification and regression tasks. For classification tasks, the overall loss is defined as:

(26) $$L=L_{cls}+\lambda_{C}L_{C}+\lambda_{T}L_{Topo}+\lambda_{M}L_{Motif}$$

where $L_{cls}$ denotes the task-specific classification loss, corresponding to the Cross-Entropy loss for binary classification and the Binary Cross-Entropy with Logits (BCEWithLogits) loss for multi-label classification. The remaining terms $L_{C}$, $L_{Topo}$, and $L_{Motif}$ denote the cross-level contrastive loss, topology-preservation loss, and motif-consistency loss, respectively. For regression tasks, the optimization objective is formulated as:

(27) $$L=L_{MSE}+\lambda_{C}L_{C}+\lambda_{T}L_{Topo}+\lambda_{M}L_{Motif}$$

where $L_{MSE}$ denotes the Mean Squared Error (MSE) regression loss between the predicted and ground-truth molecular property values, while $L_{C}$, $L_{Topo}$, and $L_{Motif}$ retain the same definitions as in the classification framework.

## 5. Experimental Setup

### 5.1. Benchmark Datasets and Data Splitting

To evaluate the performance of the proposed method, seven widely used molecular datasets from the MoleculeNet benchmark were employed [51]. These datasets include binary classification, multi-label classification, and regression, providing a comprehensive assessment of the proposed framework across various molecular property prediction tasks. Table 3 summarizes the features of all the benchmark datasets. The Blood–Brain Barrier Penetration (BBBP) [64] and β-site Amyloid Precursor Protein Cleaving Enzyme (BACE) [65] datasets were used for binary classification. The BACE dataset focuses on identifying inhibitors of the enzyme β-secretase, a key therapeutic target for Alzheimer’s disease. The Clinical Toxicity (ClinTox) [66] and SIDER [67] datasets were used to assess multi-label classification performance. ClinTox comprises clinically relevant toxicity data and can be used to predict toxicity observed in clinical trials. SIDER is a large dataset of adverse drug reactions, with several labels for each drug, and can be used to assess multi-label molecular property prediction.

Regression performance was evaluated on the Estimated Solubility (ESOL) [68], Free Solvation (FreeSolv) [69], and Lipophilicity (Lipo) [70] datasets. ESOL predicts aqueous solubility, FreeSolv estimates hydration free energy, and Lipophilicity predicts the octanol/water partition coefficient, an important physicochemical property in drug discovery. The SIDER dataset is a multi-label molecular property prediction benchmark with 27 independent adverse drug reaction labels. Each label was treated as a separate binary classification task. Accordingly, the prediction layer uses independent sigmoid outputs with the Binary Cross-Entropy with Logits (BCEWithLogits) loss rather than a Softmax classifier. The model was optimized using the standard BCEWithLogits objective without additional class re-weighting or balanced sampling. Model performance was evaluated using the mean Area Under the Receiver Operating Characteristic Curve (ROC-AUC) computed across all valid prediction tasks. For labels containing only one class in the evaluation split, ROC-AUC cannot be computed reliably; therefore, these tasks were assigned the default random-performance value when calculating the overall average, following the implementation used in our experiments. Since ROC-AUC is a threshold-independent evaluation metric, no fixed decision threshold was used for reporting the main performance results. The following benchmark datasets were split using a standard scaffold split of 80% training, 10% validation, and 10% testing. This ensures a fair and consistent experimental evaluation, and the same data partitioning strategy was used for all the datasets.

The chosen datasets span a variety of molecular properties, including permeability prediction, enzyme inhibition, clinical toxicity, adverse drug reactions, aqueous solubility, hydration free energies, and molecular lipophilicity. This diversity allows for a thorough assessment of TMGL across various molecular property prediction tasks and demonstrates its ability to learn a robust and transferable molecular representation.

### 5.2. Experimental Settings

To ensure the reliability and reproducibility of the experimental results, all experiments were conducted under a unified experimental protocol. Hyperparameter tuning was performed exclusively on the training and validation sets, while the independent test set remained unseen throughout model development to prevent information leakage and ensure an unbiased performance evaluation. Following the standard evaluation protocol adopted in MoleculeNet, all benchmark datasets were partitioned using the scaffold split strategy with an 80:10:10 ratio for the training, validation, and test sets, respectively. TMGL was implemented in PyTorch 2.11.0+cu128 and PyTorch Geometric 2.8.0.post1 with CUDA acceleration. All experiments were conducted on Google Colaboratory using an NVIDIA Tesla T4 GPU and an Intel Xeon CPU, with approximately 14.56 GB of GPU memory. The experiments used Python 3.13.15 and RDKit 2026.03.6. The same computational environment and experimental configuration were used across all benchmark datasets to ensure fair comparisons.

Model optimization used the AdamW optimizer with an initial learning rate of $1\times 10^{-4}$ and weight decay of $1\times 10^{-5}$. A cosine annealing learning-rate scheduler gradually reduced the learning rate during training, yielding more stable optimization and improved convergence. All models were trained for 300 epochs with a batch size of 32. The hidden embedding dimension was fixed at 256. The Multi-Scale GIN encoder consisted of three graph encoding layers, while the topology-aware graph transformer employed eight multi-head attention heads. ReLU was used as the activation function throughout the network. Based on empirical evaluation, the dropout rate was set to 0.30 for classification datasets and 0.05 for regression datasets. The weighting coefficients of the auxiliary objectives were fixed at $\lambda_{C}=0.001$, $\lambda_{T}=0.05$, and $\lambda_{M}=0.05$ for all experiments. The complete experimental configuration was kept identical across all benchmark datasets to ensure a fair and consistent comparison of the proposed framework with existing state-of-the-art methods.

### 5.3. Evaluation Metrics

To comprehensively evaluate the performance of the TMGL, different evaluation metrics are used depending on the prediction task. AUC-ROC is used for binary, multi-label, and graph classification datasets. AUC-ROC evaluates how well a model separates positive and negative samples across various decision thresholds. Unlike classification accuracy, AUC-ROC provides a more accurate evaluation of a molecular benchmark dataset, especially when the dataset is imbalanced, and a balance between True Positive Rate (TPR) and False Positive Rate (FPR) is required. TPR and FPR are plotted on a Receiver Operating Characteristic (ROC) curve. A quantity is defined as:

(28) $$TPR=\frac{TP}{TP+FN}$$

(29) $$FPR=\frac{FP}{FP+TN}$$

TP, TN, FP, and FN represent the counts of true positives, true negatives, false positives, and false negatives, respectively. The AUC-ROC score is the area under the ROC curve; higher values indicate a more discriminative model and better classification performance. The expected AUC ranges from 1.0 for perfect classification to 0.5 for random guessing.

The prediction accuracy for regression data is measured by Root Mean Square Error (RMSE), which is the average of the prediction errors between the predicted and observed values. RMSE is more sensitive to large errors than absolute-error-based metrics and is suitable for molecular property prediction problems where large errors are not preferred. The RMSE is calculated as:

(30) $$RMSE=\sqrt{\frac{1}{N}\sum_{i=1}^{N}{\left(y_{i}-{\hat{y}}_{i}\right)}^{2}}$$

where *N* denotes the total number of samples, $y_{i}$ represents the true target value, and ${\hat{y}}_{i}$ denotes the corresponding prediction generated by the model. Lower RMSE values indicate more accurate regression performance and better predictive capability.

### 5.4. Ablation Study Design

To evaluate the contribution of each major component in the proposed TMGL framework, an ablation study was conducted on five representative MoleculeNet benchmark datasets, including three classification datasets (BACE, ClinTox, and SIDER) and two regression datasets (ESOL and FreeSolv). For the classification datasets, the ROC-AUC was adopted as the evaluation metric, whereas RMSE was used for the regression datasets. Higher ROC-AUC and lower RMSE indicate better predictive performance. To assess the contribution of each component, a series of model variants were built by removing one module at a time from the complete TMGL framework while keeping all other network components and training settings fixed. The ablation variants exclude, respectively, topology feature extraction, motif representation learning, multi-level contrastive learning, topology preservation loss, motif consistency loss, and learned feature fusion.

## 6. Conclusions

Learning molecular representations that effectively capture both structural and semantic information is essential for accurate molecular property prediction. Although recent graph neural networks and graph transformers have achieved promising performance, several challenges remain, including the effective integration of local structural information, higher-order molecular semantics, global graph dependencies, and explicit topological information within a unified learning framework. To address these challenges, this work proposes TMGL, a unified molecular graph learning framework that combines topology feature extraction, motif representation learning, Multi-Scale GIN encoding, a topology-aware graph transformer, multi-level contrastive learning, topology preservation, motif consistency, and attention-based graph pooling to learn discriminative molecular representations in an end-to-end manner. The proposed framework was evaluated on seven widely used MoleculeNet benchmark datasets covering binary classification, multi-label classification, and regression tasks. Experimental results demonstrate that TMGL consistently outperforms existing state-of-the-art graph neural network and graph transformer models across most benchmark datasets. Furthermore, the ablation study confirms that each major component, including topology-aware feature extraction, motif representation learning, multi-level contrastive learning, topology preservation, motif consistency, and learned feature fusion contributes to the overall predictive performance. The collaborative optimization of these complementary components enables the framework to learn richer and more discriminative molecular representations for both classification and regression tasks. Future work will focus on improving the efficiency and scalability of motif extraction and topology-aware representation learning, extending the framework to larger and more diverse molecular datasets, and exploring self-supervised pretraining strategies to further improve representation quality. Investigating more efficient graph transformer architectures may also help scale TMGL to larger molecular property prediction settings.

## Figures and Tables

**Figure 1:**
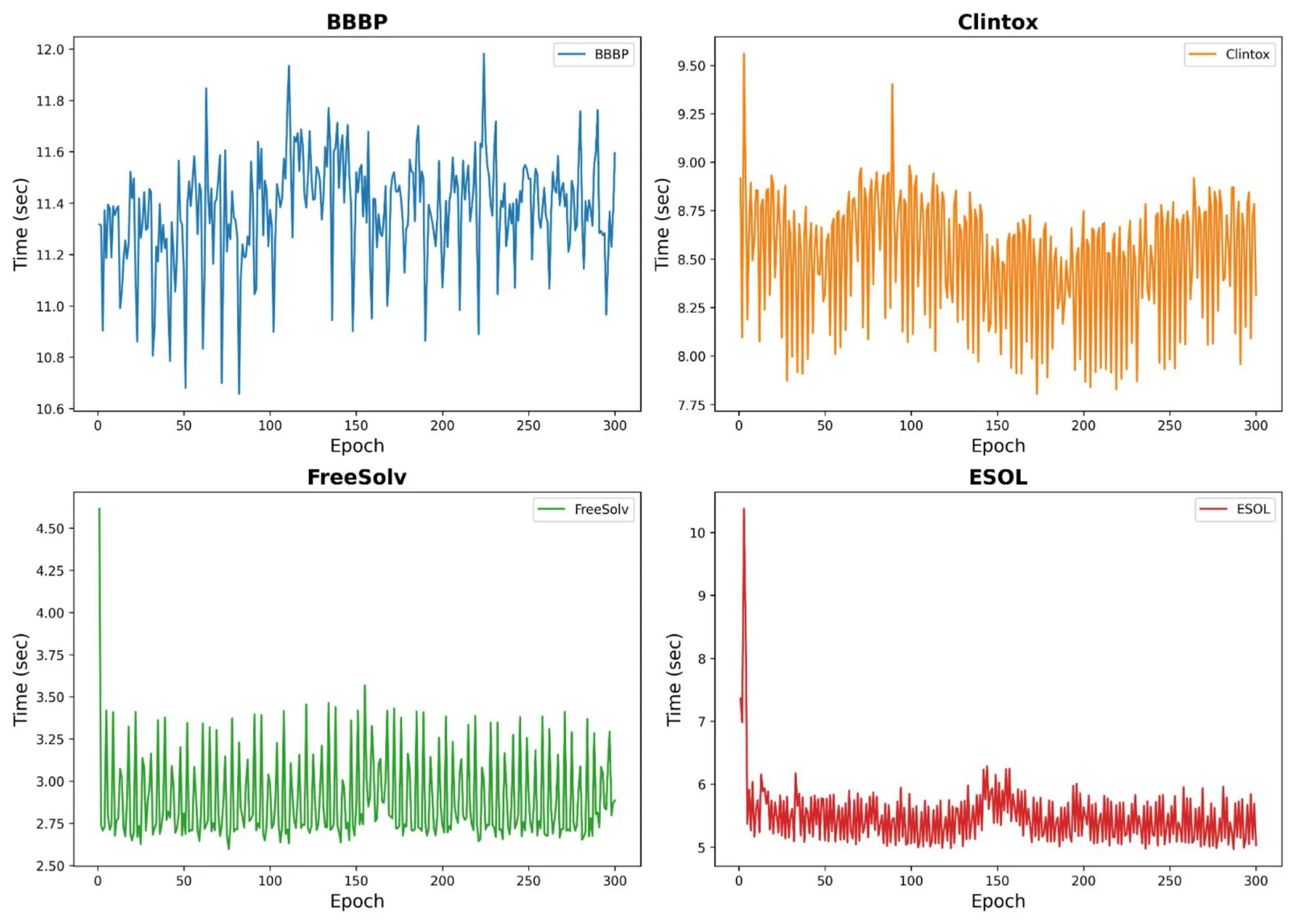
Per-epoch training time of the proposed TMGL framework across representative classification and regression datasets.

**Figure 2:**
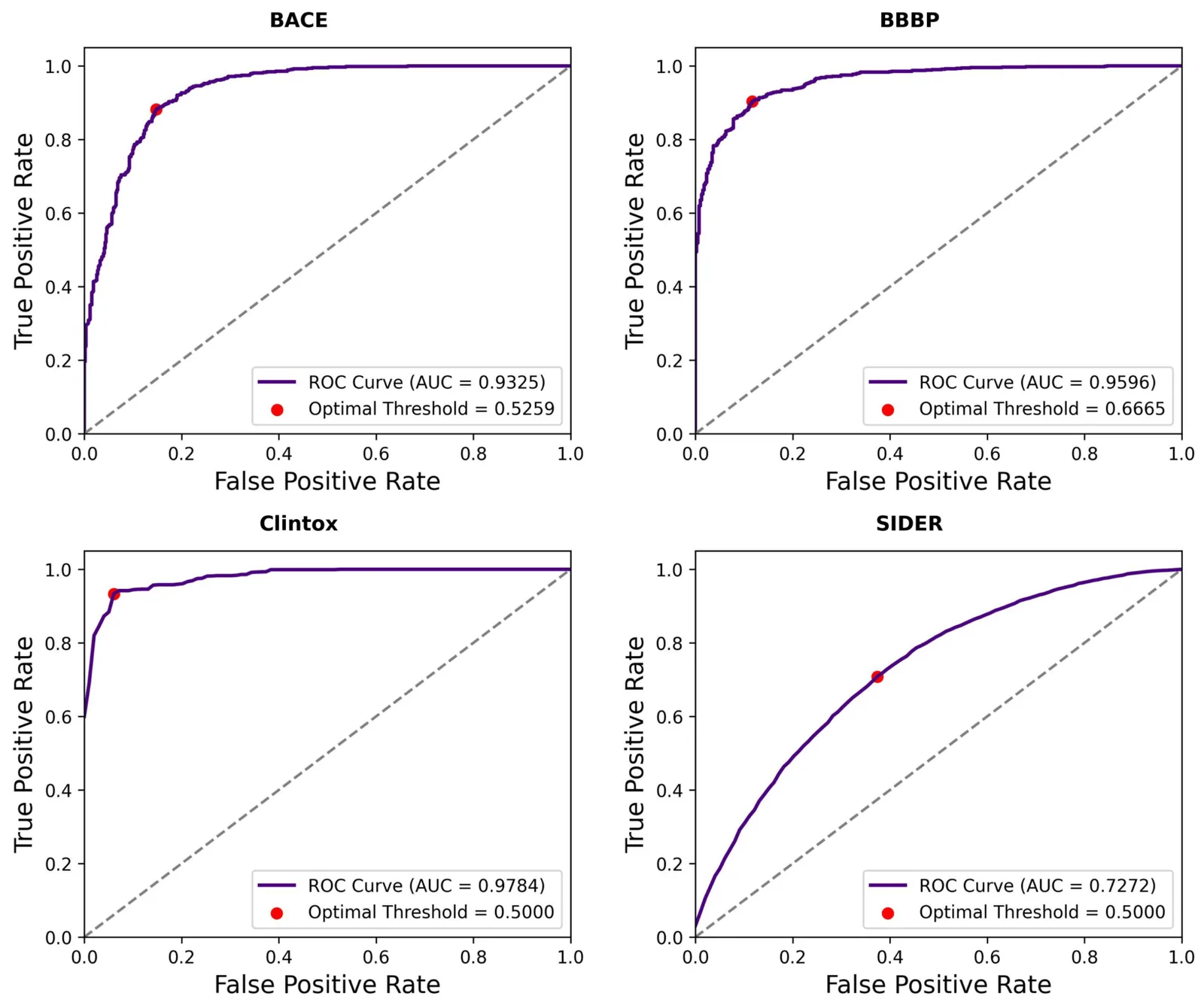
Receiver operating characteristic curves for TMGL on the BACE, BBBP, ClinTox, and SIDER benchmark datasets. Curves closer to the upper-left corner indicate stronger discrimination; the dashed diagonal represents random performance, and the red point marks the selected operating threshold in each panel.

**Figure 3:**
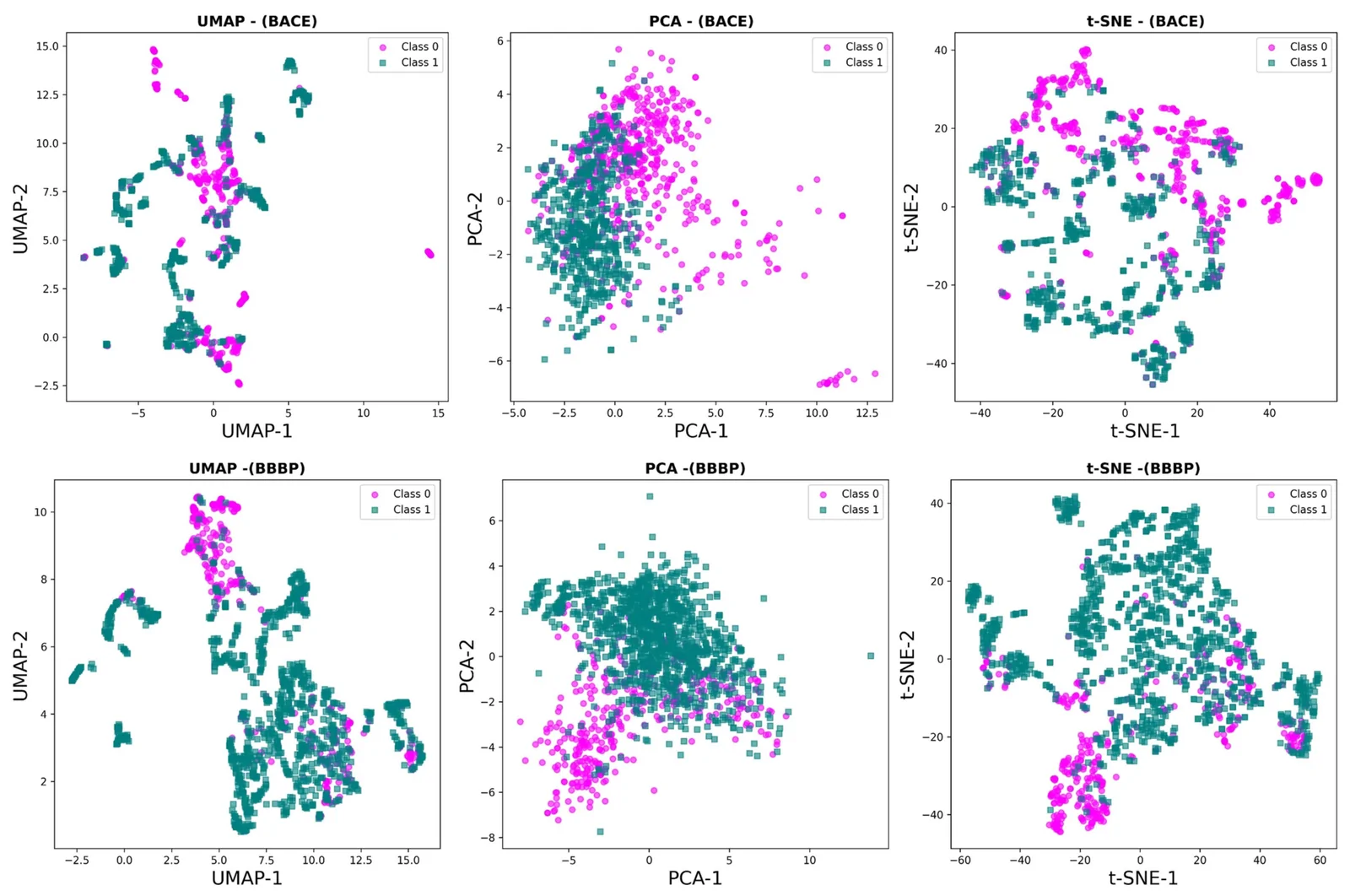
Two-dimensional TMGL embeddings for BACE (**top row**) and BBBP (**bottom row**), visualized using UMAP, PCA, and t-SNE. Points represent molecules and colors indicate class labels, revealing class separation and local clustering in the learned representation space.

**Figure 4:**
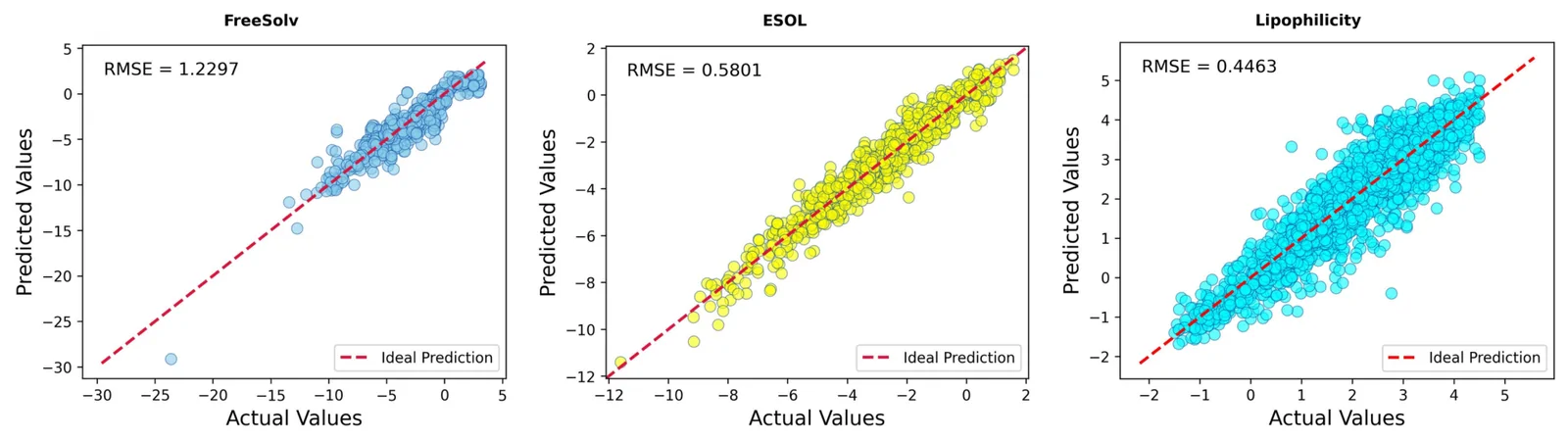
Predicted versus actual values for FreeSolv, ESOL, and Lipophilicity. Each point represents a test sample, and the dashed diagonal line indicates ideal agreement. Tighter clustering around the line indicates lower regression error and greater predictive consistency.

**Figure 5:**
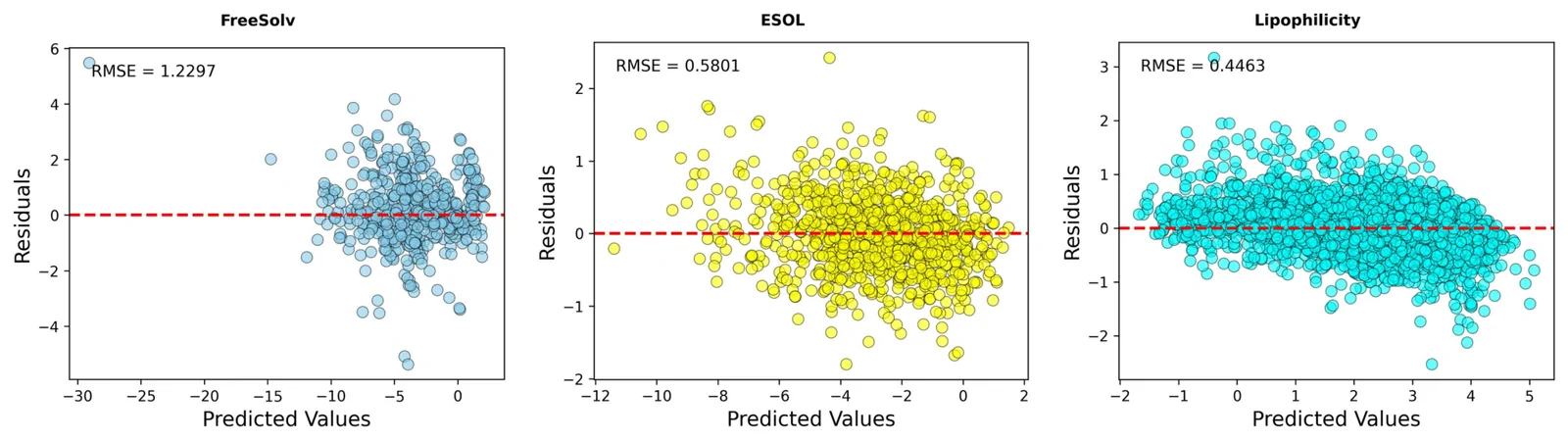
Residuals (the difference between the actual and predicted values) plotted against TMGL predictions for FreeSolv, ESOL, and Lipophilicity. Scattering around the horizontal zero line indicates limited systematic bias, while the wider FreeSolv range reflects greater variability in prediction error.

**Figure 6:**
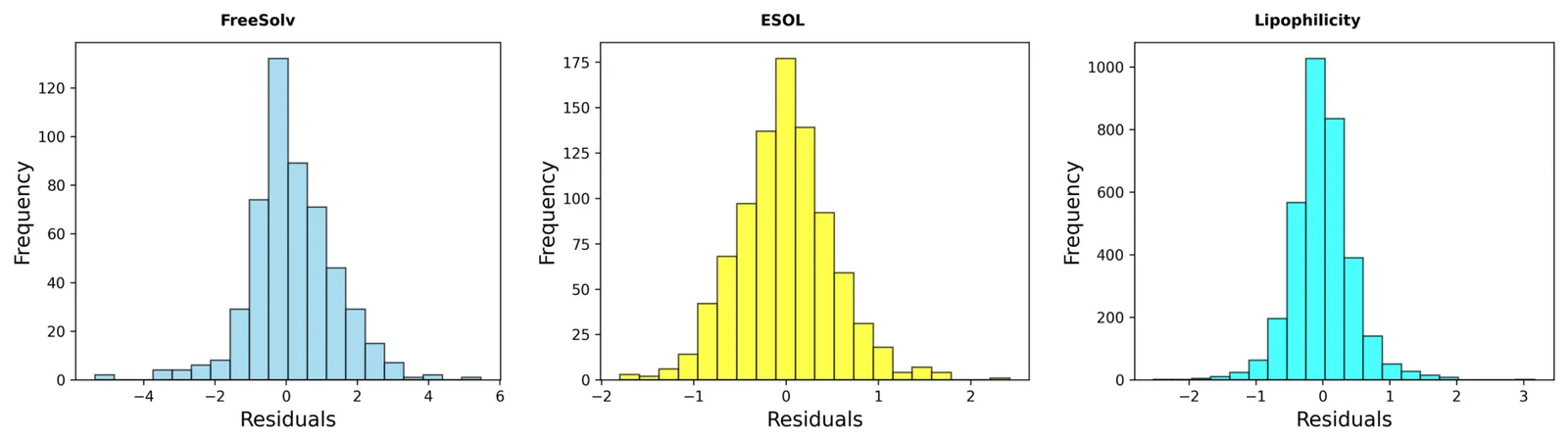
Residual histograms for FreeSolv, ESOL, and Lipophilicity. Distributions centered near zero indicate limited systematic bias; ESOL and Lipophilicity are narrower and more concentrated, whereas FreeSolv exhibits a broader prediction-error distribution.

**Figure 7:**
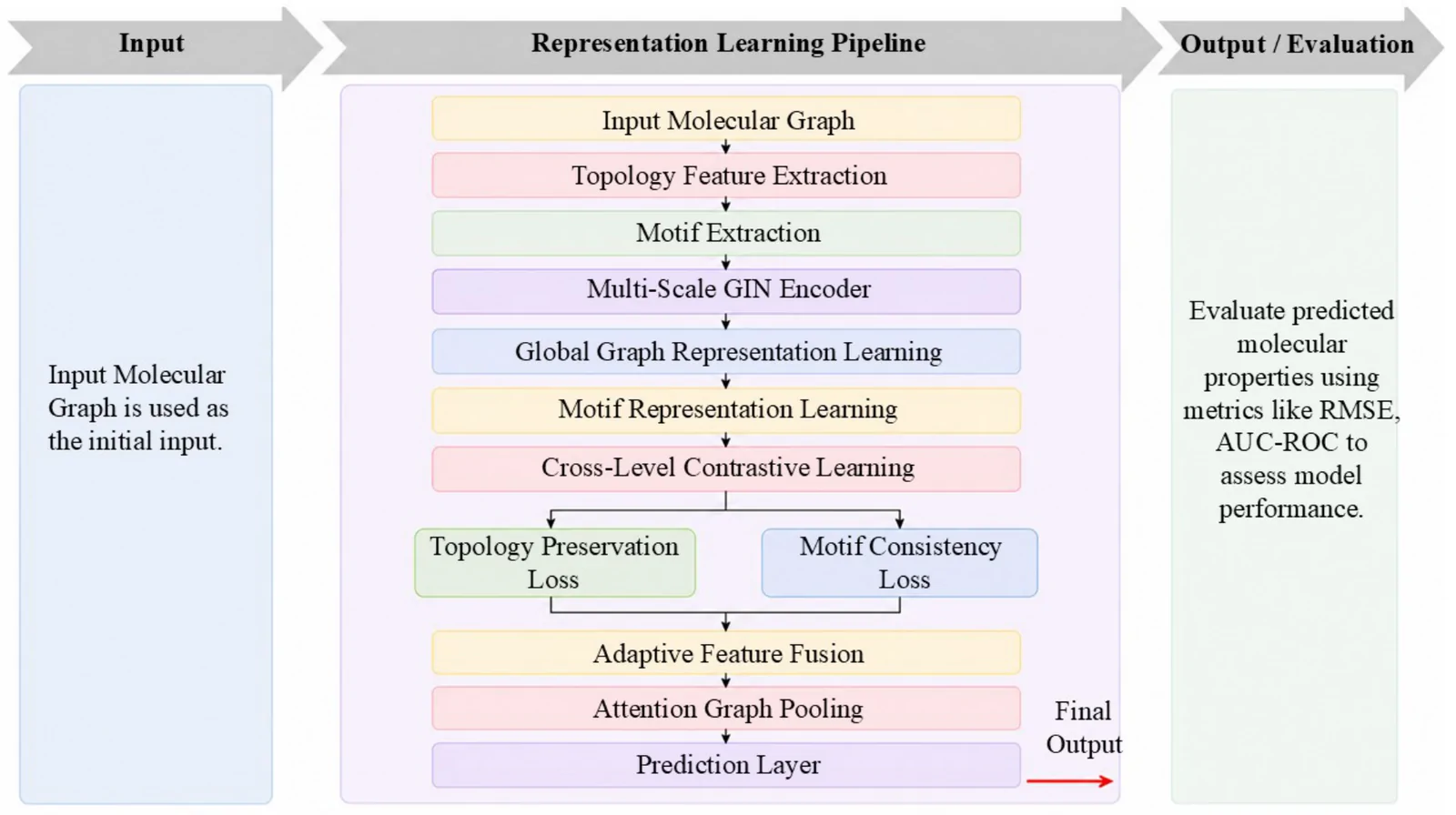
Overview of the TMGL workflow for multi-level molecular graph learning. The input graph undergoes topology and motif extraction, local and global encoding, cross-level contrastive learning, structural regularization, adaptive fusion, graph pooling, prediction, and task-specific performance evaluation.

**Figure 8:**
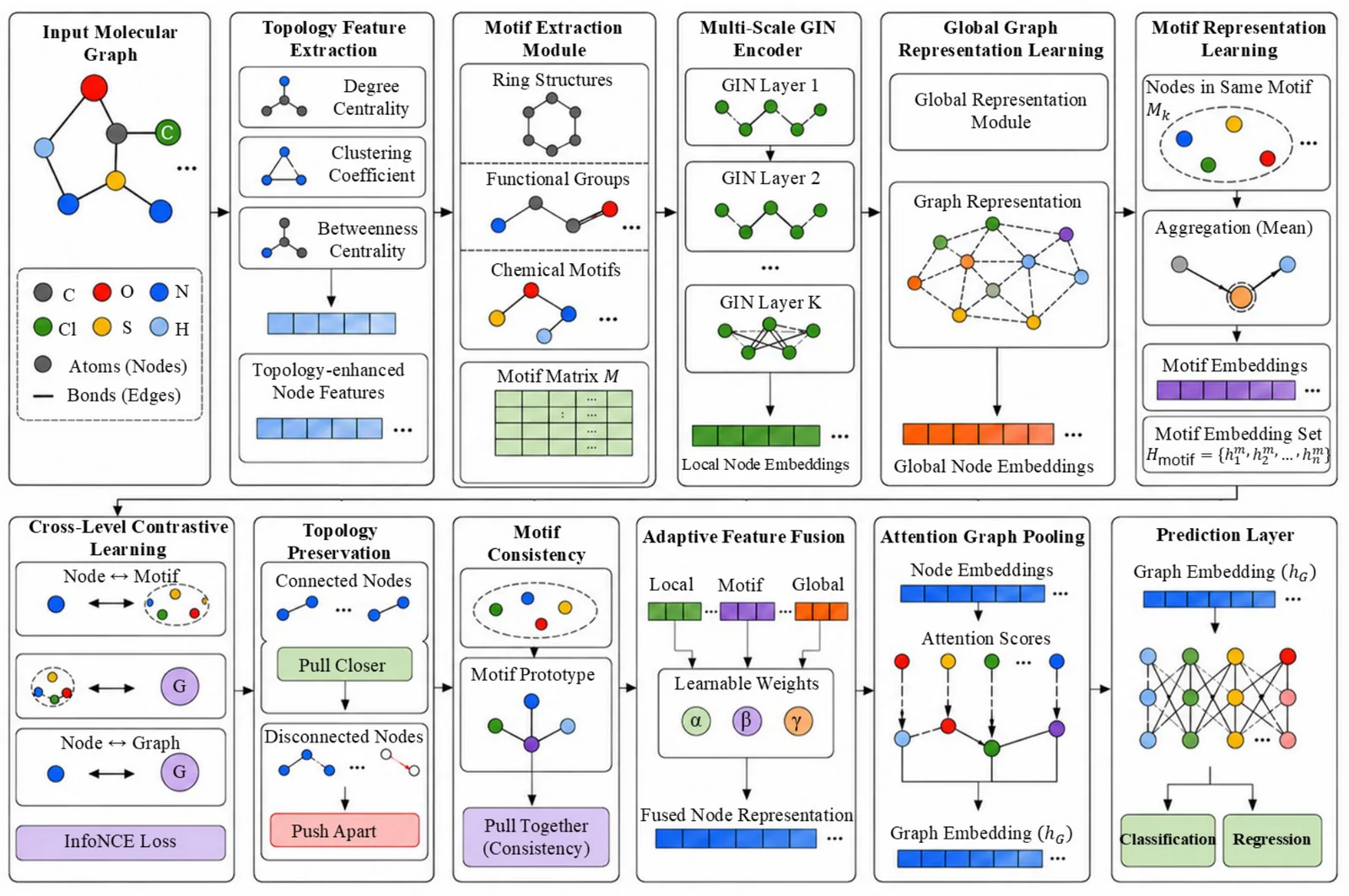
Detailed architecture of TMGL, integrating topology-enhanced node features, motif extraction, Multi-Scale GIN encoding, global Graph Transformer representations, cross-level contrastive learning, topology and motif regularization, adaptive feature fusion, attention-based graph pooling, and classification or regression prediction.

**Figure 9:**
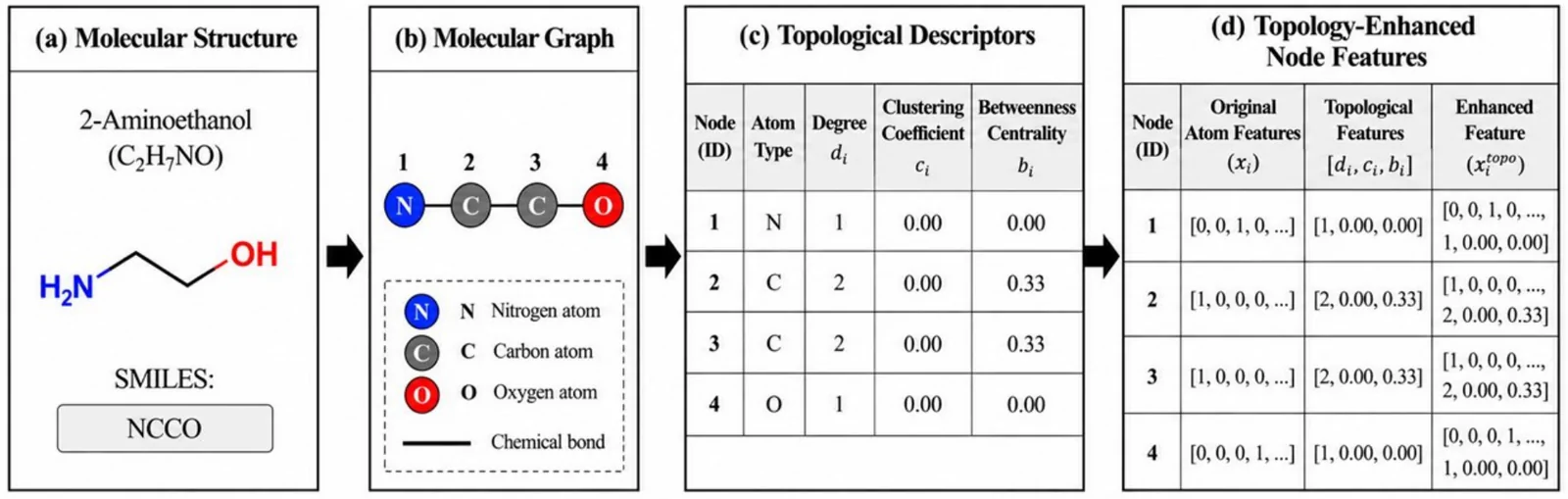
Representation of the process of topology feature extraction of a molecular structure. Topology-enhanced node representations are then created by combining the resulting degree centrality, clustering coefficient and betweenness centrality values with the original atom features.

**Figure 10:**
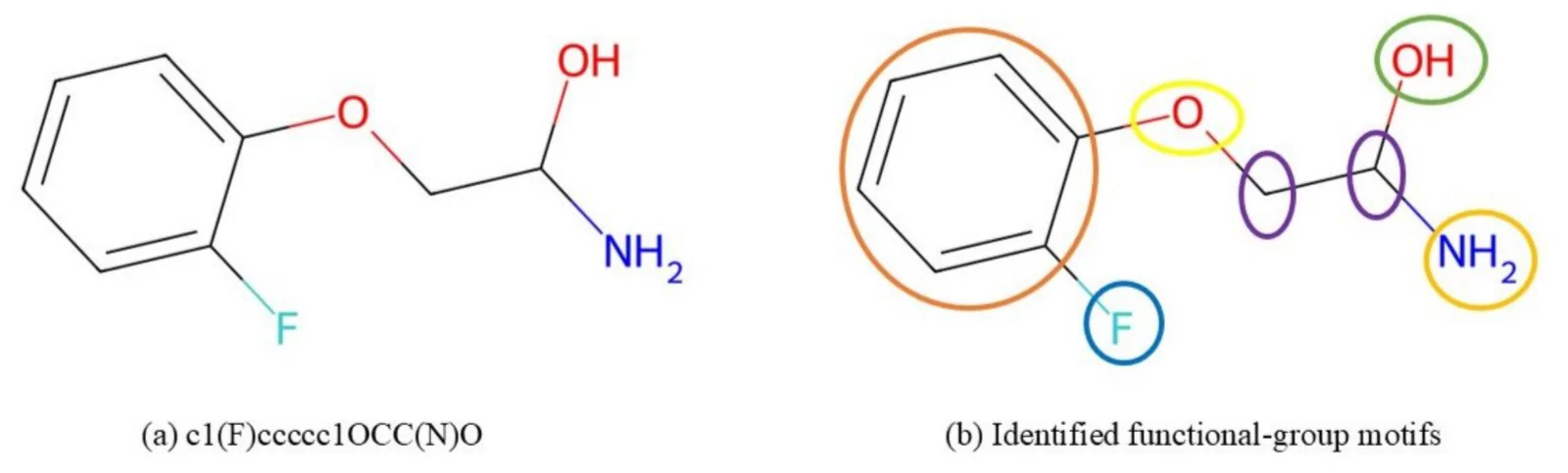
Illustration of chemical motif extraction from an atom-level molecular graph and identification of chemically meaningful substructures.

**Table 1:** Performance comparison of the proposed TMGL model and baseline methods on benchmark datasets for molecular classification (AUC-ROC ↑) and regression (RMSE ↓). Values are reported as mean ± standard deviation.

Model	Classification (AUC-ROC ↑)				Regression (RMSE ↓)		
	BACE	BBBP	ClinTox	SIDER	FreeSolv	ESOL	Lipo
SchNet [50]	0.750 ± 0.033	0.847 ± 0.024	0.717 ± 0.042	0.545 ± 0.038	3.215 ± 0.755	1.045 ± 0.064	0.909 ± 0.098
GraphConv [51]	0.854 ± 0.011	0.877 ± 0.036	0.845 ± 0.051	0.593 ± 0.035	2.900 ± 0.135	1.068 ± 0.050	0.712 ± 0.049
DMPNN [4]	0.852 ± 0.053	0.919 ± 0.030	0.897 ± 0.040	0.632 ± 0.023	2.177 ± 0.914	0.980 ± 0.258	0.653 ± 0.046
MPG [52]	0.839 ± 0.052	0.935 ± 0.012	0.915 ± 0.023	0.628 ± 0.020	2.286 ± 0.389	0.908 ± 0.105	0.637 ± 0.044
KANO [53]	0.852 ± 0.034	0.939 ± 0.016	0.938 ± 0.022	0.626 ± 0.009	1.938 ± 0.288	0.840 ± 0.150	0.596 ± 0.036
HiMol [2]	0.843 ± 0.003	0.732 ± 0.008	0.808 ± 0.014	0.613 ± 0.005	2.283	0.833	0.708
AutoML [54]	0.840 ± 0.013	0.928 ± 0.013	0.875 ± 0.058	0.682 ± 0.017	1.230 ± 0.220	0.843 ± 0.062	0.792 ± 0.043
GROVER [17]	0.894 ± 0.028	0.940 ± 0.019	0.944 ± 0.021	0.658 ± 0.023	1.544 ± 0.397	0.831 ± 0.120	0.560 ± 0.035
MGSSL [56]	0.791 ± 0.009	0.697 ± 0.009	0.807 ± 0.021	0.618 ± 0.008	—	—	—
Molformer [55]	0.881 ± 0.014	0.918 ± 0.008	0.943 ± 0.014	0.605 ± 0.011	1.145 ± 0.017	0.848 ± 0.013	0.740 ± 0.012
ProtoMol [57]	0.914 ± 0.003	0.903 ± 0.006	0.843 ± 0.003	0.681 ± 0.005	1.522 ± 0.044	0.629 ± 0.014	0.583 ± 0.039
AMCT [34]	0.922 ± 0.010	0.937 ± 0.014	—	0.641 ± 0.011	1.062 ± 0.014	0.801 ± 0.013	0.672 ± 0.011
MolGraph-xLSTM [58]	0.869 ± 0.016	0.959 ± 0.006	0.904 ± 0.032	0.697 ± 0.022	**1.024 ± 0.076**	**0.527 ± 0.046**	0.550 ± 0.026
**TMGL (Our)**	**0.937 ± 0.006**	**0.960 ± 0.016**	**0.962 ± 0.014**	**0.723 ± 0.012**	1.293 ± 0.071	0.611 ± 0.050	**0.436 ± 0.006**

*Note: Bold values indicate the best performance among all compared methods for the corresponding dataset. Higher AUC-ROC and lower RMSE values indicate better performance.*

**Table 2:** Ablation study of the proposed TMGL framework on representative molecular property prediction datasets. Higher AUC values (↑) and lower RMSE values (↓) indicate better performance.

Model Variant	BACE (AUC ↑)	ClinTox (AUC ↑)	SIDER (AUC ↑)	ESOL (RMSE ↓)	FreeSolv (RMSE ↓)
TMGL w/o Topology Features	0.907 ± 0.007	0.939 ± 0.009	0.633 ± 0.032	0.676 ± 0.018	2.198 ± 0.026
TMGL w/o Motif Representation Learning	0.894 ± 0.008	0.928 ± 0.010	0.663 ± 0.018	0.719 ± 0.020	1.556 ± 0.029
TMGL w/o Multi-Level Contrastive Learning	0.914 ± 0.006	0.946 ± 0.008	0.622 ± 0.071	0.748 ± 0.017	1.381 ± 0.023
TMGL w/o Topology Preservation Loss	0.901 ± 0.006	0.892 ± 0.007	0.691 ± 0.012	0.639 ± 0.016	1.417 ± 0.021
TMGL w/o Motif Consistency Loss	0.891 ± 0.005	0.918 ± 0.006	0.703 ± 0.011	0.645 ± 0.014	1.378 ± 0.020
TMGL w/o Learned Feature Fusion	0.929 ± 0.005	0.936 ± 0.006	0.693 ± 0.034	0.676 ± 0.014	1.337 ± 0.019
**TMGL (Full)**	**0.937 ± 0.006**	**0.962 ± 0.014**	**0.723 ± 0.012**	**0.611 ± 0.050**	**1.293 ± 0.071**

*Note: Bold values indicate the best performance among all compared methods for the corresponding dataset. Higher AUC-ROC and lower RMSE values indicate better performance.*

**Table 3:** Summary of benchmark datasets used for evaluation.

Dataset	Samples	Tasks	Task Type	Evaluation Metric
BBBP	2039	1	Binary Classification	ROC-AUC
BACE	1513	1	Binary Classification	ROC-AUC
ClinTox	1478	2	Multi-Label Classification	ROC-AUC
SIDER	1427	27	Multi-Label Classification	ROC-AUC
ESOL	1128	1	Regression	RMSE
FreeSolv	642	1	Regression	RMSE
Lipo	4200	1	Regression	RMSE

Abbreviation: RMSE, Root Mean Square Error.

## Data Availability

The data underlying the results presented in this study are publicly available. The benchmark molecular datasets were obtained from the scikit-fingerprints dataset repository (https://scikit-fingerprints.readthedocs.io/stable/modules/datasets.html, accessed on 22 September 2026). After canonicalizing SMILES strings with RDKit, the molecules were converted into graph objects with atom- and bond-level features; no additional preprocessing was applied beyond removing invalid SMILES strings. The source code and supporting files required to reproduce the results presented in this study are publicly available at https://github.com/malswadi/TMGL accessed on 22 September 2026.
